# Mediation effect of obesity on the association between triglyceride‐glucose index and hyperuricemia in Chinese hypertension adults

**DOI:** 10.1111/jch.14405

**Published:** 2021-12-13

**Authors:** Jin Sun, Mingyan Sun, Yongkang Su, Man Li, Shouyuan Ma, Yan Zhang, Anhang Zhang, Shuang Cai, Bokai Cheng, Qiligeer Bao, Ping Zhu, Shuxia Wang

**Affiliations:** ^1^ Medical School of Chinese PLA Beijing China; ^2^ Department of Geriatrics the second Medical Center & National Clinical Research Center for Geriatric Diseases, Chinese PLA General Hospital Beijing China; ^3^ Department of Ninth Health the second Medical Center & National Clinical Research Center for Geriatric Diseases, Chinese PLA General Hospital Beijing China; ^4^ Department of Geriatric Cardiology the second Medical Center, Chinese PLA General Hospital Beijing China; ^5^ Department of Outpatient the first Medical Center, Chinese PLA General Hospital Beijing China

**Keywords:** hypertension, hyperuricemia, mediation effect, obesity, triglyceride‐glucose index

## Abstract

The triglyceride glucose (TyG) index was regarded as a simple surrogate marker of insulin resistance (IR). It is confirmed that IR was significantly associated with hyperuricemia, and obesity was the risk factor for IR and hyperuricemia. However, the relationship between the TyG index and hyperuricemia and the potential role of obesity in Han Chinese hypertension are not entirely elucidated. A community‐based cross‐sectional study was conducted in 4551 hypertension patients aged 40–75 years with clinical and biochemical data. The TyG index was calculated as ln [fasting triglyceride (mg/dl) × fasting plasma glucose (mg/dl)/2]. Hyperuricemia was determined as serum uric acid ≥357μmol/L (6 mg/dl) for females and ≥417μmol/L (7 mg/dl) for males. Our study suggested that the TyG index was higher in patients with hyperuricemia than in those without (8.99±0.61, 8.70±0.59, *p* < .001). The prevalence of hyperuricemia in patients with the lowest (≤8.32), second (8.33–8.66), third (8.67–9.07) and the highest quartile (≥9.08) of the TyG index was 6.0%, 10.4%, 15.4%, 21.4%, respectively. Logistic regression analysis suggested that the higher quartile of TyG index was associated with increased hyperuricemia risk whether in crude or adjusted models (*p* < .05). Mediation analysis showed that all of our obesity indexes partially mediated the association between the TyG index and hyperuricemia to some extent. In Conclusions, the TyG index is significantly associated with hyperuricemia in hypertension patients among Han Chinese, obesity plays a partial mediation role in this relationship.

## 1 INTRODUCTION

Hyperuricemia is caused by abnormal purine metabolism, including excessive uric acid production or insufficient renal excretion, which is one of the components of metabolic syndrome. Previous studies have shown that hyperuricemia is associated with the occurrence and development of many metabolic disorders and cardiovascular diseases. For example, hypertensive patients with hyperuricemia occurred more cardiovascular events than those without hyperuricemia.^1^, ^2^ Recently epidemiological study reported that there are 170 million patients with hyperuricemia in China,^3^ which greatly increases the morbidity and mortality of cardiovascular events. Therefore, it is very important to optimize the risk stratification method of hyperuricemia to identify people at high risk of cardiovascular events and other complications. Some studies have reported that IR and obesity are independent risk factors for hyperuricemia, which are significantly related to the occurrence and development of hyperuricemia.^4^, ^5^

It is recognized that the bidirectional correlation between IR and hyperuricemia.^6^, ^7^ The increase of uric acid level will lead to the impairment of endothelial function, which in turn reduces insulin sensitivity by reducing the bioavailability of nitric oxide, and eventually leads to IR.^8^, ^9^ In contrast, IR induces hyperuricemia by increasing the reabsorption of uric acid and sodium in renal tubules.^10^ Some epidemiological studies have also confirmed the two‐side effect of IR and hyperuricemia. A longitudinal study reported the unidirectional association between the two. They found that hyperuricemia leads to IR, then partially mediates the development of hypertension.^11^ But another study found that the improvement of insulin sensitivity reduces the level of serum uric acid.^12^ In short, IR and hyperuricemia promote each other to form a vicious circle, resulting in more serious organ damage. In addition, some studies confirmed that IR is related to obesity,^13^ and obesity is a major risk factor for hyperuricemia,^14^ so the correlation between IR and hyperuricemia may be partially or completely mediated by obesity.

At present, the TyG index is considered as an alternative index to identify IR,^15^ and it is confirmed that the correlation with the occurrence and prognosis of many IR‐related diseases.^16^ It has been reported that there is a positive correlation between the TyG index and the risk of hyperuricemia,^17^, ^18^, ^19^ and the IR was more significant in patients with hypertension complicated with hyperuricemia. However, most of these studies were conducted in relatively healthy communities. The role of the TyG index in assessing the risk of hyperuricemia in hypertensive patients and the potential mechanism of obesity in it have not been fully clarified. Therefore, the purpose of our study is to explore the relationship between TyG index and the prevalence of hyperuricemia in the hypertensive population, and further to clarify whether obesity indexes (BMI, WC, HC) play an intermediary role in it, to optimize the risk stratification method of hyperuricemia and further identify people at very high risk of cardiovascular events and other complications in a hypertensive population.

## 2 METHODS

### 2.1 Study population

This was a community‐based cross‐sectional study, and participants were recruited from the Xinyang county, in the middle region in China from 2004 to 2005. We used a multistage cluster sample method to select a representative sample of rural community residents aged 40–75 years. A total of 13 444 patients (5270 men and 8174 women) were incorporated into the survey, which was from 63 districts of Xinyang's seven residential communities and yielding a response rate of 84.9%. Among them, 5421 hypertensive patients were identified and thoroughly examined. Hypertension was defined as diastolic blood pressure (DBP) of ≥90 mm Hg, SBP of ≥140 mm Hg, physician diagnosis, or current medication for hypertension (as defined by WHO 1999). Of 5421 hypertensive patients, 4805 patients had complete echocardiographic data, and 254 patients were excluded because of no data about other clinical characteristics or blood biochemical indexes. Ultimately, 4551 patients (1531 men and 3020 women) with integrated clinical data remained in the present study.

### 2.2 Clinical characteristics

Based on the age on the residence documents, we identified those who are eligible participants, then we invited them by letter or phone to the community clinic. All the participants were interviewed and required to complete a standardized questionnaire that included general information, such as sex, age, medical history, lifestyle behaviors, and so on. Anthropometric measurement was performed by experienced research staff with uniform instruments. The height and weight of all participants were measured in the upright position with light clothing and bare feet, and the error range is not more than 0.1 cm or 0.1 kg. We measured waist and hip measurements for all participants, also while standing. The Blood pressure was measured using a standard official protocol. Systolic and diastolic blood pressures (SBP, DBP) were measured using the portable Doppler device (ES‐101EX, HADECO, 8 MHz probes, Kawasaki, Japan) and standard 12‐cm cuff in each arm with the patients resting for at least 5 min. The Doppler Stethoscope was placed at the humeral artery fluctuation, quickly inflate the cuff to 20–30 mm Hg above the palpated SBP, and then deflated at the rate of 2–6 mm Hg/s. The first sound heard is the SBP of the brachial artery, which continues to deflate until the sound disappears or suddenly becomes weak, the scale indicated by the mercury column is DBP. The average of three readings with the participant in the sitting position after at least 5 min of rest, recorded at least 30s apart, was obtained for analysis.

Transthoracic echocardiography was performed according to standard protocols, under the supervision of two ultrasound physicians with at least 2 years of experience, and performed by two technicians trained in echocardiography at the Institute of Cardiology, Chinese Academy of Medical Sciences. The patients respiring quietly in the left decubitus position, and the echocardiographic indicators were measured at the end of systolic and end‐diastolic periods of up to three cardiac cycles, including left atrium diameter, diastolic left ventricular inner diameter (LVIDD), diastolic left ventricular posterior wall thickness (PWTd), diastolic interventricular septal thickness (IVSd), E wave deceleration time, transmitted E wave velocity and transmitted A wave velocity. Then we calculated the left ventricular mass (LVM) and left ventricular mass index (LVMI) based on the above data. LVM was calculated by using the equation: 0.8×1.04 ((IVSd + LVIDD + PWTd)^3^—LVIDD^3^) + 0.6. LVMI was calculated by dividing LVM by height^2.7^(LVMI_h2.7_).

 BMI was calculated as the ratio of the weight in kg divided by the square of the height in m. Glomerular filtration rate (GFR) was used to reflect renal function, which was calculated by the Cockcroft‐Gault formula: GFR (ml/min) = [(140‐ age)×body weight (kg)×(0.85 female)]/72×serum creatinine (mg/dl). The diagnosis of diabetes mellitus (DM) was based on an increased fasting plasma glucose (≥7.0 mmol/L), previous physician diagnosis, or current anti‐diabetic medication. The diagnosis of stroke was based on the results of strict neurological examination, computed tomography, or magnetic resonance imaging tests, which were verified from local hospital records. Coronary heart disease (CAD) was diagnosed by the results of coronary arteriography, a previous myocardial infarction, or surgery or coronary revascularization.

### 2.3 Biochemical parameters

Fasting blood samples were obtained from an antecubital vein of participants after overnight fasting. Serum was separated from the blood samples by centrifuged on‐site. Then the serum samples were delivered to the Beijing center laboratory on the dry ice for analysis. The fasting plasma glucose (FPG), triglycerides (TG), total cholesterol (TC), high‐density lipoprotein (HDL) cholesterol, low‐density lipoprotein (LDL) cholesterol, uric acid, and other blood biochemical indices were quantified enzymatically by an automatic analyzer (Hitachi 7060, Hitachi, Tokyo, Japan).

The TyG index was calculated as ln [fasting TG (mg/dl) ×FPG (mg/dl)/2].^20^ Patients were divided into four groups according to the TyG index quartile: Q1 (TyG≤8.32), Q2 (8.33≤TyG≤8.66), Q3 (8.67≤TyG≤9.07), and Q4 (≥9.08). Hyperuricemia was determined as serum uric acid ≥357μmol/L (6 mg/dl) for females and ≥417μmol/L (7 mg/dl) for males.^21^

### 2.4 Statistical analysis

Data management and statistical analysis were performed using SPSS 22.0 for Windows (SPSS Inc, Chicago, IL, USA). Data are reported as the mean ± standard deviation for continuous variables and as percentages for categorical variables. Continuous variable independent sample t‐test and classified variable chi‐square test were used for the differences between hyperuricemia group and non‐hyperuricemia group. All participants were stratified by quartiles of TyG index, baseline differences in clinical variables between groups using analysis of variance (ANOVA) for continuous variables, and Chi‐squared test for categorical variables. We used the multivariable‐adjusted logistic regression model to evaluate the relationship between TyG index categories and hyperuricemia, and the values of odds ratios (ORs) and 95% confidence intervals (CIs) were calculated. Sequential models were developed to minimize the effect of confounders. Model 1 was a crude model. Model 2 was adjusted for age, sex, SBP, DBP, serum creatinine, blood urea nitrogen (BUN), GFR, history of stroke, CAD, and DM. Model 3 was adjusted for all variables of model 2 plus serum TC, HDL‐C, LDL‐C BMI, HC, and WC.

**FIGURE 1:**
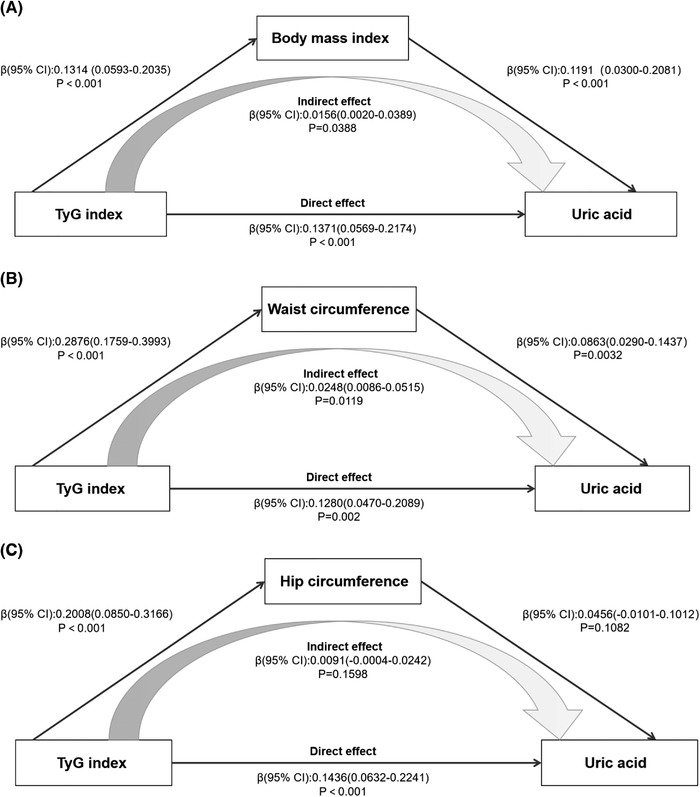
Mediation effect to BMI (A) or WC (B) or HC (C) on the relationship between TyG index and uric acid in the hyperuricemia group. The parameter estimate of total effect is 0.1528(0.0730–0.2325), *p*<.001. Adjusted for age, sex, systolic blood pressure, diastolic blood pressure, serum creatinine, blood urea nitrogen, glomerular filtration rate, the history of stroke, coronary artery disease and diabetes mellitus, serum cholesterol, high‐density lipoprotein cholesterol, low‐density lipoprotein cholesterol

To further examine the impact of BMI, WC, and HC on the association between TyG index and hyperuricemia, we constructed mediation models for analysis in the whole population, hyperuricemia, and non‐hyperuricemia. In the model, TyG index is predictor, BMI, HC, and WC are mediators respectively, uric acid is the outcome, and the confounders in model 3 were adjusted in the mediation analysis. The simplified mediation model is presented in Figure 1, which involved several main paths as follows. Path a: the association between TyG index and obesity indexes; Path b: the association of obesity indexes with uric acid; Path c and Path c’: the total and direct effects of TyG index on uric acid, respectively. In addition, Path ab mains the indirect effect of the TyG index on uric acid, and the sum of direct and indirect effects equals the total effect. The obesity indexes played a complete mediating role in the association between the TyG index and uric acid when the total effect and indirect effect are significant but the direct effect is not significant. The obesity indexes were the incomplete mediator when the direct effect is also significant. In addition, when the indirect effect was not significant, it indicated that the obesity index did not mediate the correlation between TyG and uric acid. The differences were considered significant if a two‐tailed *p* value < .05.

## 3 RESULTS

### 3.1 Clinical characteristics of patients by hyperuricemia

In the whole group of 4551 hypertension patients, there were 605 patients with hyperuricemia, the prevalence of hyperuricemia was 13.29%. The clinical characteristics of the study population by hyperuricemia are described in Table 1. The average age of all participants was 58.63±8.33 years, of which patients with hyperuricemia were 60.11±8.76 years, and it was 58.41±8.24 years in people without hyperuricemia. Males comprised 33.6% of the total participants, 51.2% of the hyperuricemia group, and 30.9% of the non‐hyperuricemia group. Compared to patients without hyperuricemia, those with hyperuricemia were more likely to be older, with a higher proportion of males, had higher weight, height, BMI, WC, HC, serum TG, TC, HDL‐C, LDL‐C, uric acid, BUN, and creatinine, and more CAD status and stroke history (all *p*<.05). In addition, Echocardiographic indicators (IVSd, PWTd, LVM, and LVMI) were significantly higher in the hyperuricemia group than in the non‐hyperuricemia group. Particularly worth mentioning is the TyG index is significantly higher in patients with hyperuricemia than without it (8.99±0.61, 8.70±0.59, *p*<.001).

**TABLE 1:** Clinical characteristics of participants by hyperuricemia

Variables	Total	Hyperuricemia	Non‐hyperuricemia	*p* value
Age (year)	58.63±8.33	60.11±8.76	58.41±8.24	<.001
Male	1531(33.6%)	310(51.2%)	1221(30.9%)	<.001
Height (cm)	157.70±7.94	160.23±8.64	157.31±7.75	<.001
Weight (Kg)	65.37±14.40	69.80±11.22	64.69±14.71	<.001
BMI (kg/m^2^)	26.23±5.06	27.14±3.53	26.10±5.24	<.001
WC (cm)	85.45±12.09	89.68±11.92	84.80±11.98	<.001
HC (cm)	98.25±10.95	100.72±10.80	97.87±10.93	<.001
SBP (mm Hg)	163.50±24.47	165.11±25.66	163.26±24.28	.084
DBP (mm Hg)	97.04±12.62	98.18±12.88	96.86±12.57	.017
Glucose (mmol/L)	5.57±1.69	5.57±1.20	5.56±1.75	.947
Triglyceride (mmol/L)	1.68±1.24	2.17±1.52	1.61±1.17	<.001
Cholesterol (mmol/L)	5.53±1.10	5.84±1.19	5.48±1.08	<.001
HDL‐C (mmol/L)	1.55±0.34	1.48±0.34	1.56±0.34	<.001
LDL‐C (mmol/L)	3.15±0.86	3.37±0.93	3.12±0.84	<.001
BUN (mmol/L)	5.47±1.81	6.48±2.53	5.31±1.61	<.001
Creatinine(umol/L)	66.25±26.00	86.86±43.48	63.09±20.37	<.001
Uric acid (umol/L)	292.78±86.77	447.57±65.42	269.05±61.56	<.001
TyG index	8.73±0.60	8.99±0.61	8.70±0.59	<.001
History of stroke	467(10.3%)	83(13.7%)	384(9.7%)	.002
History of CAD	415(9.1%)	85(14%)	330(8.4%)	<.001
Diabetes mellitus	374(8.2%)	50(8.3%)	324(8.2%)	.937
Antidiabetics	52(1.1%)	9(1.3%)	43(1.1%)	.408
Lipid‐lowering agent	13(0.3%)	2(0.3%)	11(0.3%)	.688
Echocardiographic data				
IVSd (mm)	1.00±0.16	1.03±0.16	1.00±0.16	<.001
PWTd (mm)	0.97±0.14	1.00±0.14	0.97±0.13	<.001
LVM (g)	158.65±44.11	172.23±49.61	156.57±42.83	<.001
LVMIh (g/m2.7)	46.43±12.53	48.26±13.40	46.14±12.36	<.001

*Abbreviations*: BMI, body mass index; BUN, blood urea nitrogen; CAD, coronary artery disease, TyG index, triglyceride‐glucose index; DBP, diastolic blood pressure; HC, hip circumference; HDL‐C, high‐density lipoprotein cholesterol; IVSd, end‐diastolic interventricular septal thickness; LDL, low‐density lipoprotein cholesterol; LVMI_h,_ left ventricular mass index divided by height^2.7.^; PWTd, end‐diastolic posterior wall thickness; SBP, systolic blood pressure; WC, waist circumference.

### 3.2 Clinical characteristics of patients by TyG index

Based on the above results, the TyG index was grouped by quartile, and we presented the baseline characteristics of the patients in Table 2 according to the TyG index categorical. In all participants, the prevalence of hyperuricemia with the lowest, second, third, and highest quartile of TyG index were 6.0, 10.4, 15.4, 21.4%, respectively. It means an increasing trend in the prevalence of hyperuricemia with the higher TyG index (*p*<.001). Compared with patients with the lowest quartiles of TyG index, those with higher quartile of TyG index had higher weight, BMI, WC, HC, SBP, DBP, heart rate, and more CAD and DM status (all *p*<.001). In the biochemical index results, the group with a higher TyG index had higher serum FPG, TG, TC, HDL‐C, uric acid, and lower BUN, LDL‐C (all *p*<.001). There were no significant differences in age, height, serum creatinine, or the morbidity of stroke among those groups.

**TABLE 2:** Clinical characteristics of participants by TyG index

Variables	Q1 (≤8.32)	Q2 (8.33‐8.66)	Q3 (8.67‐9.07)	Q4 (≥9.08)	*p* value
N	1140	1136	1137	1138	–
Age (year)	58.52±8.78	58.76±8.52	58.90±8.08	58.35±7.93	.406
Male	474(41.6%)	375(33.0%)	342(30.1%)	340(29.9%)	<.001
Height (cm)	158.06±7.86	157.50±8.06	157.30±7.88	157.94±7.93	.074
Weight (Kg)	62.38±20.68	64.39±11.20	66.08±11.42	68.65±11.15	<.001
BMI (kg/m^2^)	24.91±7.42	25.90±3.17	26.66±3.93	27.47±3.82	<.001
WC (cm)	80.70±13.35	84.32±11.24	86.89±10.48	89.92±12.09	<.001
HC (cm)	94.12±12.89	97.84±10.18	99.71±9.26	101.32±9.78	<.001
SBP (mm Hg)	160.72±24.16	164.19±24.72	164.18±24.52	164.92±24.30	<.001
DBP (mm Hg)	95.77±12.31	97.46±13.04	97.00±12.43	97.92±12.59	<.001
Heart rate	70.74±11.28	72.06±11.70	73.97±12.65	74.51±12.99	<.001
Glucose(mmol/L)	4.90±0.67	5.20±0.72	5.46±1.00	6.71±2.75	<.001
Triglyceride (mmol/L)	0.83±0.18	1.20±0.19	1.67±0.31	3.03±1.78	<.001
Cholesterol (mmol/L)	5.05±0.90	5.36±1.00	5.70±1.05	6.02±1.18	<.001
HDL‐C (mmol/L)	1.68±0.35	1.60±0.34	1.52±0.32	1.40±0.29	<.001
LDL‐C (mmol/L)	2.80±0.71	3.09±0.80	3.36±0.87	3.36±0.92	<.001
Creatinine(umol/L)	66.05±26.27	66.67±22.48	66.65±30.68	65.63±23.84	.736
BUN (mmol/L)	5.63±1.83	5.54±1.77	5.40±1.84	5.30±1.76	<.001
Uric acid (umol/L)	267.72±75.74	286.00±81.65	300.58±87.62	316.85±93.48	<.001
History of stroke	102(8.9%)	110(9.7%)	132(11.6%)	123(10.8%)	.160
History of CAD	78(6.8%)	93(8.2%)	95(8.4%)	149(13.0%)	<.001
Diabetes mellitus	5(0.4%)	17(1.5%)	64(5.6%)	288(25.3%)	<.001
Hyperuricemia	68(6.0%)	118(10.4%)	175(15.4%)	244(21.4%)	<.001

*Abbreviations*: BMI, body mass index; BUN, blood urea nitrogen; CAD, coronary artery disease; DBP, diastolic blood pressure; HC, hip circumference; SBP, systolic blood pressure; HDL‐C, high‐density lipoprotein cholesterol; LDL, low‐density lipoprotein cholesterol; TyG index, triglyceride‐glucose index.; WC, waist circumference.

### 3.3 Association of TyG index with hyperuricemia

To explore the relationship between the TyG index and hyperuricemia, we built logistics regression models, and the results were presented in Table 3. Our study revealed a positive correlation between the TyG index and hyperuricemia. As shown in Table 3, participants with the second, third, and highest quartile of TyG index were 1.83, 2.87, and 4.30 times more likely to have hyperuricemia compared with the lowest quartile in the crude model (*p*<.001). After controlling for age, sex, SBP, DBP, serum creatinine, BUN, GFR, the history of stroke, CAD, and DM in model 2, the OR (95% CI) for hyperuricemia by TyG index groups was changed to 1.91(1.36–2.68), 3.55(2.57–4.90) and 6.29(4.56–8.68), respectively. The relationship between TyG index and hyperuricemia remained significant after further adjustment for serum TC, HDL‐C, LDL‐C, BMI, WC, and HC in model 3, and the OR (95% CI) of second, third, and highest quartile of TyG index compared with the lowest quartile were 1.62(1.15–2.28), 2.48(1.77–3.49) and 3.45(2.35–5.08), respectively. In particular, the association between the TyG index and hyperuricemia attenuated after being adjusted for several biochemical markers and BMI but remained significant.

**TABLE 3:** Odds ratio (95% CI) of hyperuricemia by triglyceride‐glucose index

	Q1 (≤8.32)		Q2 (8.33‐8.66)		Q3 (8.67‐9.07)		Q4 (≥9.08)	
Hyperuricemia	68(6.0%)		118(10.4%)		175(15.4%)		244(21.4%)	
	OR (95%CI)	*p*‐value	OR (95%CI)	*p*‐value	OR (95%CI)	*p*‐value	OR (95%CI)	*p*‐value
Model1	Reference		1.83(1.34‐2.49)	<.001	2.87(2.14‐3.85)	<.001	4.30(3.24‐5.71)	<.001
Model2	Reference		1.91(1.36‐2.68)	<.001	3.55(2.57‐4.90)	<.001	6.29(4.56‐8.68)	<.001
Model3	Reference		1.62(1.15‐2.28)	.006	2.48(1.77‐3.49)	<.001	3.45(2.35‐5.08)	<.001

*Abbreviations*: 95% CI, 95% confidence interval; OR, odds ratio.

Model 1: unadjusted.

Model 2: adjusted for age, sex, systolic blood pressure, diastolic blood pressure, serum creatinine, blood urea nitrogen, glomerular filtration rate, the history of stroke, coronary artery disease and diabetes mellitus.

Model 3: adjusted for model 2 plus serum cholesterol, high‐density lipoprotein cholesterol, low‐density lipoprotein cholesterol, body mass index, waist and hip circumference.

### 3.4 Association of TyG index with obesity indexes and serum uric acid

Table 4 shows the associations of the TyG index with obesity indexes (BMI, WC, and HC) and serum uric acid. The β coefficients (95% CI) of second, third, highest quartile of TyG index with BMI were 0.09(0.03–0.15), 0.15(0.09–0.21), and 0.22(0.15–0.28), respectively (*p* < .001) after adjusting for age, sex, SBP, DBP, serum creatinine, BUN, GFR, the history of stroke, CAD and DM. Similarly. TyG index was significantly positively correlated with WC and HC. In addition, the relationship between the TyG index and serum uric acid level is also statistically significant. Compared to the lowest quartile of the TyG index, the β coefficients (95% CI) of the second, third, highest quartile of TyG index with uric acid were increased gradually, which are 0.27, 0.48, 0.74, respectively (*p* < .001).

**TABLE 4:** The association of triglyceride‐glucose index with hyperuricemia and obesity indexes

	TyG index			
	Q1 β(95%CI);*p*‐value	Q2 β(95%CI);*p*‐value	Q3 β(95%CI);*p*‐value	Q4 β(95%CI);*p*‐value
BMI (kg/m2)	Reference	0.09(0.03‐0.15); < .001	0.15(0.09‐0.21); < .001	0.22(0.15‐0.28); < .001
WC (cm)	Reference	0.26(0.18‐0.33); < .001	0.43(0.36‐0.51); < .001	0.64(0.56‐0.72); < .001
HC (cm)	Reference	0.28(0.20‐0.35); < .001	0.41(0.33‐0.48); < .001	0.51(0.43‐0.59); < .001
Uric acid (umol/L)	Reference	0.27(0.20‐0.33); < .001	0.48(0.41‐0.54); < .001	0.74(0.67‐0.81); < .001

*Abbreviations*: 95% CI, 95% confidence interval; BMI, body mass index; HC, hip circumference.; TyG index, triglyceride‐glucose index; WC, waist circumference.

All adjusted for age, sex, systolic blood pressure, diastolic blood pressure, serum creatinine, blood urea nitrogen, glomerular filtration rate, the history of stroke, coronary artery disease, diabetes mellitus.

### 3.5 The mediating role of obesity indexes

To further examine the impact of obesity indexes on the association between the TyG index and hyperuricemia, we constructed a mediation model for analysis in the whole population, hyperuricemia, and non‐hyperuricemia. The results of mediation analysis were displayed in Table 5 and the simplified mediation model was presented in Figure 1 and Figures S1 and S2. We found that all of our interested obesity indexes had a mediation impact (to various extents) on the link between the TyG index and uric acid. In the total population, BMI, WC, and HC all partially mediated the correlation between TyG index and uric acid, the total effect TyG index on uric acid was 0.2446 (0.2057–0.2339), and the mediation proportion of 8.9%, 17.4%, and 10.3%, respectively. The results in the non‐hyperuricemia group were similar to those in the general population, but the proportion of mediators was increased (9.0%, 19.0%, and 12.0%, respectively). However, in the hyperuricemia group, BMI and WC also partially mediated this correlation, which the total effect was 0.1528(0.0730–0.2325) and the mediating proportion of 10.2% and 16.2%, respectively. But the indirect effect of HC was not significant, that is, HC did not mediate the relationship between TyG index and hyperuricemia in the hyperuricemia group.

**TABLE 5:** Mediation analysis of the relationship between triglyceride‐glucose index and uric acid level by obesity indexes

	Direct effect		Indirect effect		
Potential mediators	β (95% CI)	*p* value	β (95% CI)	*p* value	Proportion of mediation (%)
Whole group					
BMI	0.2228 (0.1844–0.2612)	<.001	0.0218 (0.0045–0.0495)	<.001	8.9%
WC	0.2021 (0.1632–0.2409)	<.001	0.0425 (0.0306–0.0582)	<.001	17.4%
HC	0.2194 (0.1806–0.2582)	<.001	0.0252 (0.0172–0.0359)	<.001	10.3%
HUA group					
BMI	0.1371(0.0569–0.2174)	<.001	0.0156(0.0020–0.0389)	.0388	10.2%
WC	0.1280(0.0470–0.2089)	.002	0.0248(0.0086–0.0515)	.0119	16.2%
HC	0.1436(0.0632–0.2241)	<.001	0.0091(–0.0004–0.0242)	.1598	–
Non‐HUA group					
BMI	0.1224(0.0911–0.1537)	<.001	0.0121(0.0003–0.0324)	<.001	9.0%
WC	0.1090(0.0773–0.1406)	<.001	0.0255(0.0158–0.0371)	<.001	19.0%
HC	0.1184(0.0867–0.1500)	<.001	0.0161(0.0096–0.0245)	<.001	12.0%

*Abbreviations*: OR, Odd ratio; BMI, body mass index; HC, hip circumference; 95%CI, 95%Confidence interval; TyG index, triglyceride‐glucose index; WC, waist circumference.

Adjusted for age, sex, systolic blood pressure, diastolic blood pressure, serum creatinine, blood urea nitrogen, glomerular filtration rate, the history of stroke, coronary artery disease and diabetes mellitus, serum cholesterol, high‐density lipoprotein cholesterol, low‐density lipoprotein cholesterol.

## 4 DISCUSSION

In this study, we observed that a strong cross‐sectional correlation between the higher TyG index and the increased prevalence of hyperuricemia in Chinese patients with hypertension, which was statistically significant even after adjusting for clinical data and risk factors for hyperuricemia. We further evaluated the mediation effect of different obesity factors on the link of TyG index and serum uric acid level, we found that higher TyG index was associated with higher obesity index (BMI, WC, HC) and serum uric acid level, while the obesity factors partially mediated the relationship between TyG index and uric acid to a varied extent.

Hyperuricemia is a common metabolic abnormality and an independent risk factor for cardiovascular disease.^22^ A meta‐analysis of 44 studies showed that the total prevalence of hyperuricemia in the Chinese community population was 13.3%,^21^ our study was included hypertensive people, but the prevalence was similar to that in community people, which is abnormal. We speculated that may be due to the deviation of the sex ratio of our participants. Previous studies have reported that the prevalence of hyperuricemia in hypertensive people is higher than that in normal people,^23^ and the prevalence of hyperuricemia in males is significantly higher than that in females (19.4% vs. 7.9%),^21^ and our participants are far fewer in males than in females (19.4% vs. 7.9%).

Hypertensive patients with hyperuricemia have a very high risk of developing cardiovascular disease.^24^ A large number of cross‐sectional and prospective studies have shown that high levels of uric acid increased the risk of target organ damage and cardiovascular events in patients with hypertension.^25^, ^26^ In our study, the prevalence of stroke and CAD in the hyperuricemia group was significantly higher than that in the normal uric acid group, and the significant differences in echocardiographic indicators between the two groups also indicated the damage of high uric acid levels to the heart. The potential mechanism involves many aspects. Due to increased blood pressure caused by renal blood flow decreased, the body compensatory to perceive less blood volume will increase heavy absorption decrease urine output, to maintain endovascular blood volume. Therefore, high blood pressure in patients with renal blood flow reduction will cause uric acid absorption increases, caused by high uric acid hematic disease occurred.^27^ In addition, the damage of high uric acid to vascular endothelial function leads to vascular dilatation dysfunction, increased vascular resistance, stimulation of vascular smooth muscle cell proliferation, resulting in vascular sclerosis, which in turn leads to increased blood pressure, forming a small vicious cycle.^28^ Hypertension is the most important risk factor for cardiovascular disease due to its damage to the function and structure of blood vessels,^29^ while hyperuricemia leads to arterial stiffness by increasing the level of oxidative stress and inflammation in the body. The synergistic effect of the two accelerates the occurrence of cardiovascular complications.^30^, ^31^ Therefore, looking for new biological indicators to identify hyperuricemia in patients with hypertension is very important for the early prevention and intervention of cardiovascular complications.

IR plays a key role in the occurrence and development of hyperuricemia. In terms of mechanism, IR inhibits uric acid excretion by increasing renal tubular sodium reabsorption, while uric acid, in turn, causes IR by reducing the bioavailability of nitric oxide, mitochondrial oxidative stress, inflammation, and other mechanisms.^32^, ^33^ In short, there is a bidirectional relationship between IR and hyperuricemia. At present, it has been reported that there are many methods to evaluate IR according to simple biochemical indexes, such as the TyG index, TG/HDL‐C, METS‐IR, and so on. The ability of the TyG index to identify metabolic disorders in the Chinese population and its applicability in different populations are significantly better than other indexes.^34^, ^35^

As a new, simple and effective substitute for IR, the correlation between the TyG index and hyperuricemia may be due to its ability to accurately predict IR. In addition, TyG index was calculated as ln (fasting triglyceride [mg/dL] × fasting blood glucose [mg/dL] / 2). Triglyceride and fasting blood glucose also affected uric acid metabolism.^6^, ^36^ A part of free fatty acids generated by triglyceride decomposition will be re‐esterified or enter other tissues, and this process will accelerate the utilization rate of ATP. Every 1 mol of triglyceride will promote the utilization of 7–8 mol of ATP. The increase of blood triglyceride will lead to the generation and utilization of more free fatty acids, thus accelerating the decomposition of ATP and leading to the increase of uric acid production.^37^ In addition, studies have also analyzed the mechanism of interaction between triglyceride and uric acid metabolism from the perspective of pentose phosphate metabolism. Phosphoric ribose and NADPH are the raw materials for purine synthesis. The hydrogen of NADOH is also the source of hydrogen needed for fat synthesis. The enhancement of pentose phosphate metabolism can promote the increase of fat synthesis, which leads to the increase of blood triglyceride, and also promotes the synthesis of purine, which further promotes the production of uric acid, which leads to the increase of blood uric acid.^38^ There is an inverted U‐shaped relationship between fasting blood glucose and uric acid level,^39^, ^40^ the level of fasting serum uric acid in prediabetic patients was higher than the normal blood glucose people, but the level of fasting serum uric acid in diabetic patients was lower,^18^, ^41^ which may be due to hyperinsulinemia associated with IR increasing uric acid levels by increasing uric acid production and/or reducing uric acid excretion.^6^ However, when fasting glucose rises to a certain threshold, elevated glucose levels in urine lead to competitive inhibition of uric acid reabsorption and increased uric acid excretion.^36^

The above mechanism has been verified by epidemiological studies. A cross‐sectional study of the Chinese general population reported that the risk of hyperuricemia increases proportionally with the increase of the TyG index. They think the TyG index can significantly improve the risk identification ability of the risk prediction model for hyperuricemia, suggesting its important value in optimizing the risk stratification of hyperuricemia.^17^ Recently, Li and coworkers found that the correlation between TyG index and the risk of hypertension with hyperuricemia is more significant than that with hyperuricemia or hypertension alone, suggesting that IR is more significant in patients with hyperuricemia with hypertension.^19^ Mazidi and coworkers also reported a significant correlation between serum uric acid levels and IR in general communities of the United States, and they further found that a variety of obesity indicators mediated this correlation in varying degrees.^18^

In addition, some studies have suggested that obesity is a prerequisite for metabolic syndrome, and IR, hyperuricemia, and hypertension are all manifestations of metabolic syndrome.^42^ Unfortunately, no studies have reported the effect of obesity on TyG index and hyperuricemia in hypertensive people, and previous studies on the role of obesity in the link between TyG index and hyperuricemia are inconsistent. In 2017, Mazidi and coworkers reported that a variety of obesity indicators mediated the relationship between TyG and serum uric acid to varying degrees, of which the regulatory ratio of BMI to WC was 46.8% and 57.1%, respectively.^18^ However, a recent study reports that the link between the TyG index and hyperuricemia is not related to the mediation of BMI in the general community population in China.^17^ Our study found that BMI, WC, and HC did partially mediate the relationship between TyG and serum uric acid, but the intermediary proportion was significantly lower than that of the NHANES study. We speculate that the reason for this difference may be due to ethnic differences and medical history differences in the included population. Previous studies have been reported that race affects insulin sensitivity and obesity‐related diseases,^43^ while the incidence of overweight and IR in patients with hypertension is significantly higher than that in healthy people,^44^ suggesting that IR and obesity indicators are also affected by hypertension. Perhaps for the above reasons, our study shows that the intermediary role of obesity in Chinese hypertensive people, which is different from that of the general population in China and weaker than that of the American population. It is worth mentioning that our study found that HC did not mediate the relationship between TyG index and uric acid in people with hyperuricemia, suggesting that the mediating effect of HC is only applicable to low‐risk people.

The incidence of cardiovascular events and metabolic diseases in patients with hyperuricemia is significantly higher than that in healthy people, and hypertension is a common risk factor and complication of hyperuricemia. At present, there are 245 million people with hypertension and 170 million patients with hyperuricemia in China. Early identification of high‐risk groups of hyperuricemia in patients with hypertension and early intervention for patients with complications is very important for the prevention and treatment of cardiovascular disease. Our study observed that a higher TyG index is significantly associated with a higher prevalence of hyperuricemia in hypertensive people. Therefore, the TyG index may be used as a monitoring indicator of hyperuricemia, which is helpful to formulate prevention and intervention strategies.

This study has several limitations to consider. First of all, our study only included the hypertensive population of the Han nationality in rural China, which could not represent the urban population. Second, the proportion of men and women included in this study is uneven, and men are far less than women, which may be because a large number of male rural residents leave home to work in cities. However, previous studies have reported that the incidence of hyperuricemia in men is significantly higher than that in women,^21^ so follow‐up studies with a more balanced sex ratio may be needed to prove our point of view. Third, there is a lack of information about uric acid and antihypertensive drugs in our study. Due to the early study design, we did not obtain data on the usage of uric acid drugs, which may have an unknown effect on hyperuricemia, but considering that many previous studies did not report the use of hypouricemia drugs in the investigation of hyperuricemia,^10^, ^17^, ^18^ we believe our results are still acceptable. Fourth, there is a high correlation (collinearity) between WC, HC, and BMI, so the mediating effect of WC and HC may be affected by BMI, and vice versa. However, it is not feasible to include two mediations in the same model to test the above problems. Therefore, this study may underestimate the relationship between obesity and uric acid. In addition, although a high correlation between WC, HC and BMI, there are still differences between these three indicators. BMI is the most commonly used indicator of obesity in clinical practice and epidemiological studies, which takes into account factors such as height and weight, simple and convenient. WC and HC reflect the regional distribution of fat, and WC is a widely used indicator of central obesity.^45^ Therefore I think our study is still meaningful. Finally, this is a cross‐sectional study, so that we can only observe the correlation between the TyG index, hyperuricemia, and obesity indicators, but not know their time sequence. More rigorous prospective studies are needed to support our conclusions.

## 5 CONCLUSIONS

Our results show that a higher TyG index is significantly associated with the prevalence of hyperuricemia in hypertensive people. We further proved this link may be partially mediated by obesity and discussed the differences in the mediating effects of obesity in different populations. Therefore, as a non‐invasive and low‐cost indicator of non‐insulin‐dependent IR, the TyG index may become a potential monitoring index in the treatment of hyperuricemia and better identify high‐risk patients in people with traditional risk factors, because it can further identify the extremely high‐risk population of hyperuricemia in patients with hypertension, and then contribute to the formulation of prevention and intervention strategies. Our study quantifies the role of obesity in the association between TyG index and uric acid, and the control of obesity may contribute to the primary prevention of IR and hyperuricemia. In summary, we recommend routine determination of the TyG index in hypertensive people, which is a simple, low‐cost but very beneficial test. In addition to screening patients with IR, it is also helpful to identify hypertensive people with hyperuricemia, who with a high risk of cardiovascular complications.

## CONFLICT OF INTEREST

All the authors declared that they have no conflict of interest.

## AUTHORS' CONTRIBUTIONS

J.S. contributed to data interpretation, critical review of the manuscript, drafting the manuscript, and revising the comment of reviewers. M.S. and Y.S. contributed to manuscript preparation, analysis tools and assist with data analysis. M.L., S.M., and Y.Z. contributed to manuscript revisions. A.Z., S.C., B.C., and Q.B. helped with data analysis. S.W. contributed significantly to data collection, the conception of the study, and revising the comment of reviewers. P.Z. contributed to the conception of the study and helped perform the analysis with constructive discussions.

## Supporting information

Supporting material
