# Supplementary material for: Mediation effect of obesity on the association between triglyceride‐glucose index and hyperuricemia in Chinese hypertension adults
Source: J Clin Hypertens (Greenwich). 2021 Dec 13;24(1):47–57. doi: 10.1111/jch.14405 (PMC8783353; doi:10.1111/jch.14405)
Supplement: Supplementary file 1 — Supporting material [file JCH-24-47-s001.docx]

**
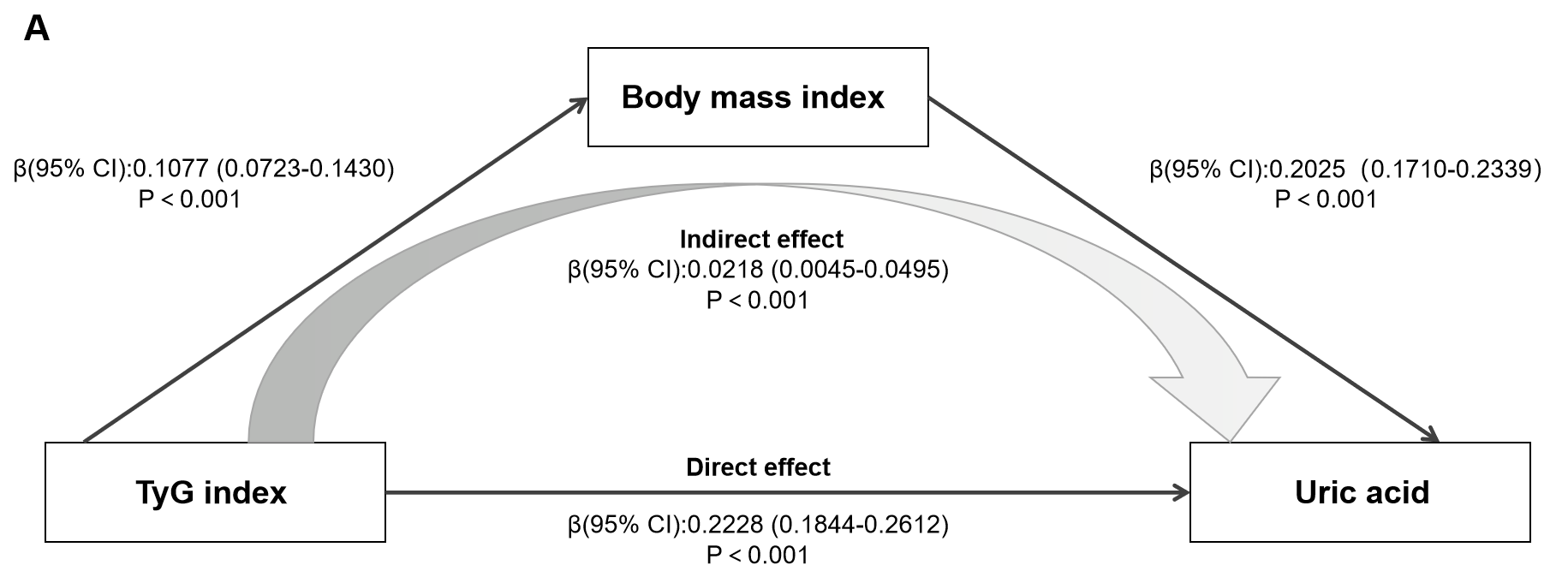

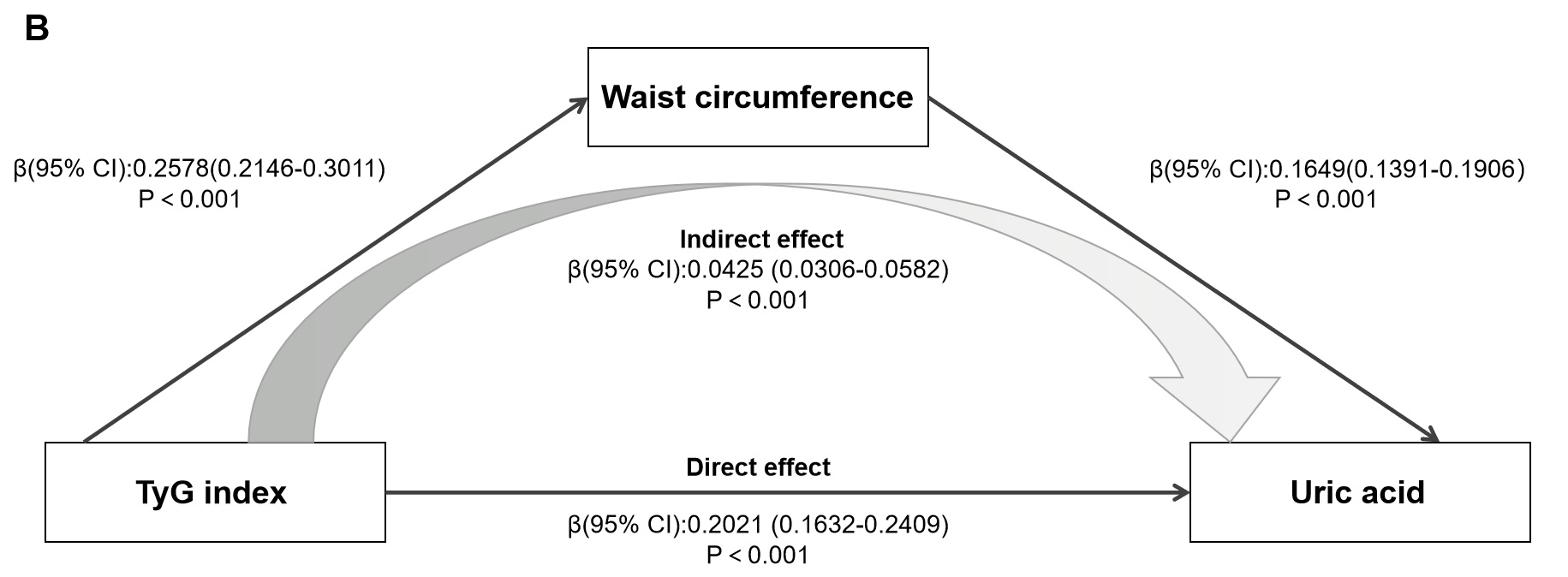

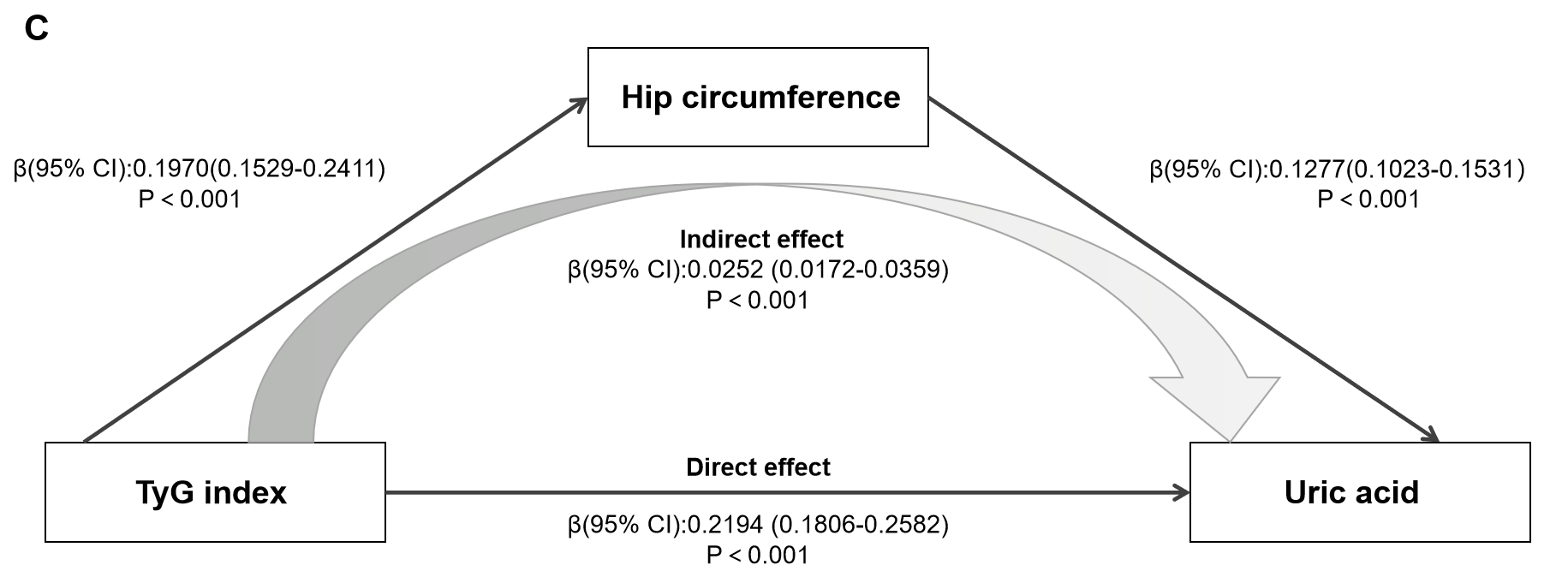
**

**Supplement Figure 1** Mediation effect to BMI **(A)** or WC **(B)** or HC **(C)** on the relationship between TyG index and uric acid in the whole group. The parameter estimate of total effect is 0.2446(0.2057-0.2835), P＜0.001. Adjusted for age, sex, systolic blood pressure, diastolic blood pressure, serum [creatinine](F:/Dict/8.9.9.0/resultui/html/index.html#/javascript:;), [blood urea nitrogen](F:/Dict/8.9.9.0/resultui/html/index.html#/javascript:;), [glomerular](F:/Dict/8.9.9.0/resultui/html/index.html#/javascript:;) [filtration](F:/Dict/8.9.9.0/resultui/html/index.html#/javascript:;) [rate](F:/Dict/8.9.9.0/resultui/html/index.html#/javascript:;), the history of stroke, coronary artery disease and diabetes mellitus, serum cholesterol, high-density lipoprotein cholesterol, low-density lipoprotein cholesterol.


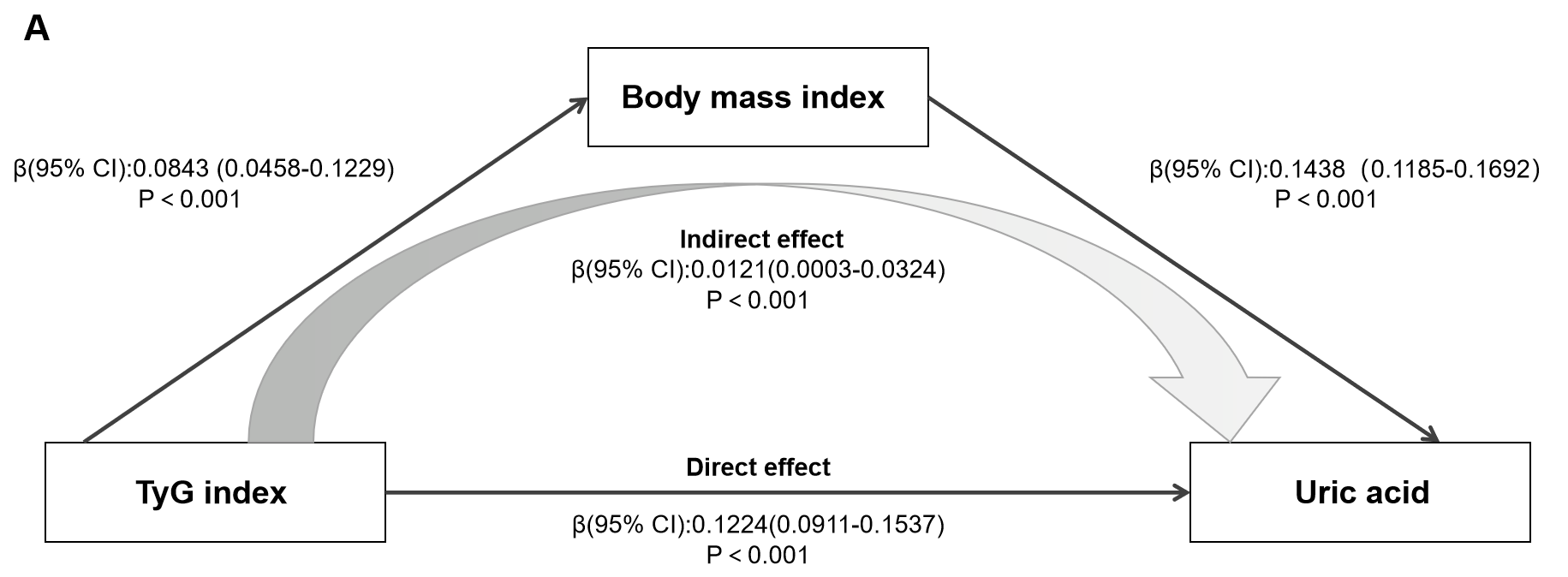

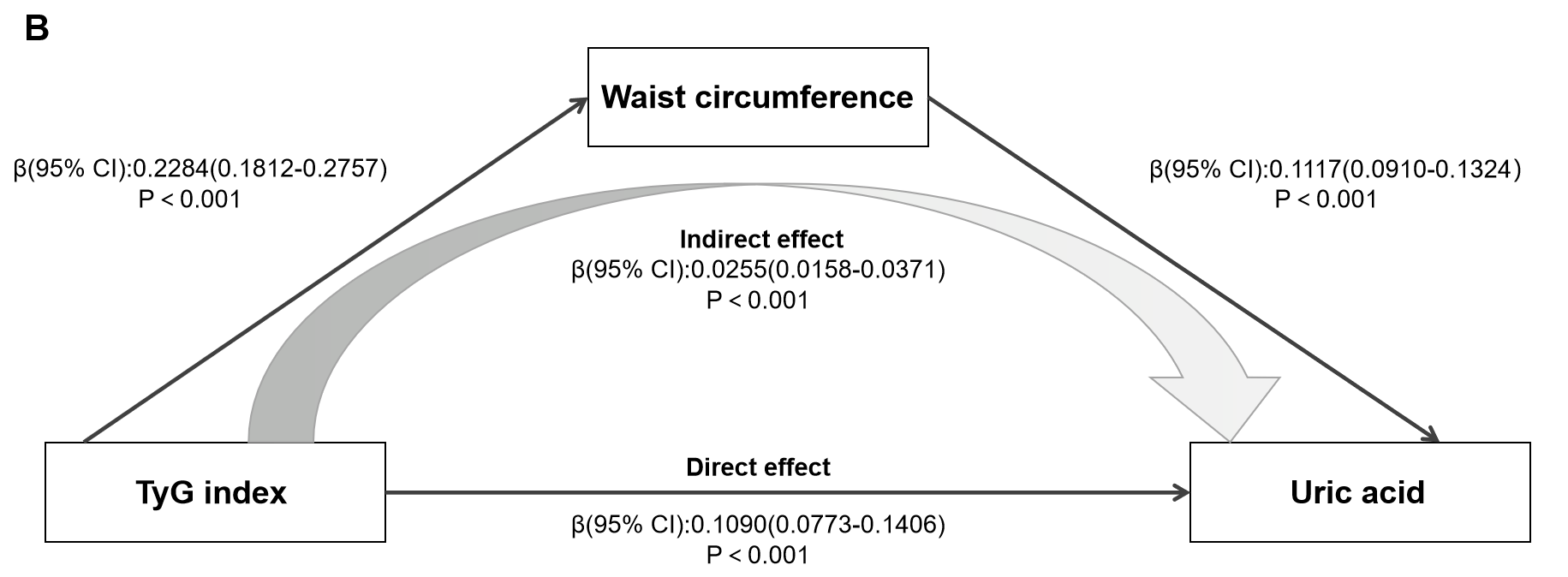

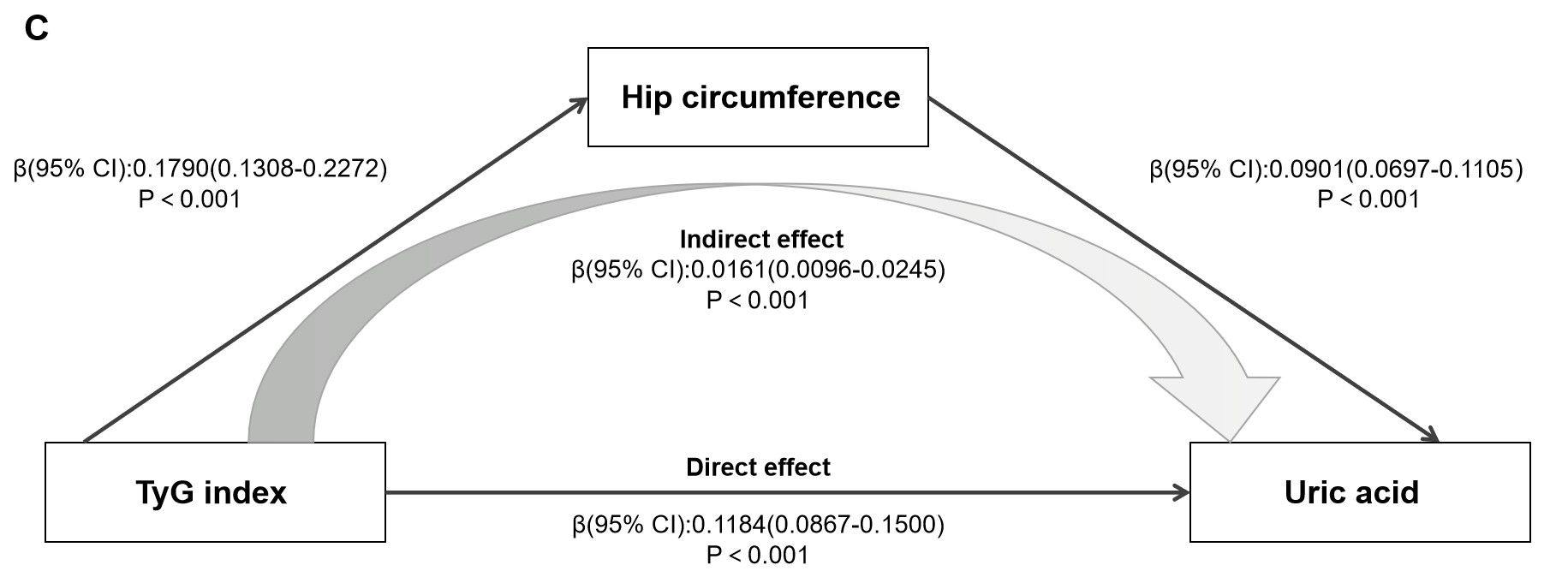


**Supplement Figure 2** Mediation effect to BMI **(A)** or WC **(B)** or HC **(C)** on the relationship between TyG index and uric acid in the non-hyperuricemia group. The parameter estimate of total effect is 0.1345(0.1028-0.1662), P＜0.001. Adjusted for age, sex, systolic blood pressure, diastolic blood pressure, serum [creatinine](F:/Dict/8.9.9.0/resultui/html/index.html#/javascript:;), [blood urea nitrogen](F:/Dict/8.9.9.0/resultui/html/index.html#/javascript:;), [glomerular](F:/Dict/8.9.9.0/resultui/html/index.html#/javascript:;) [filtration](F:/Dict/8.9.9.0/resultui/html/index.html#/javascript:;) [rate](F:/Dict/8.9.9.0/resultui/html/index.html#/javascript:;), the history of stroke, coronary artery disease and diabetes mellitus, serum cholesterol, high-density lipoprotein cholesterol, low-density lipoprotein cholesterol.
